# Microwave Assisted Suzuki-Miyaura and Ullmann Type Homocoupling Reactions of 2- and 3-Halopyridines Using a Pd(OAc)_2_/Benzimidazolium Salt and Base Catalyst System

**DOI:** 10.3390/molecules18043712

**Published:** 2013-03-25

**Authors:** Ülkü Yılmaz, Selma Deniz, Hasan Küçükbay, Nihat Şireci

**Affiliations:** 1 Battalgazi Vocational School, İnönü University, Battalgazi, Malatya 44210, Turkey; E-Mail: ulku.yilmaz@inonu.edu.tr; 2 Department of Elementary Education, Faculty of Education, Hakkari University, Hakkari 30000, Turkey; E-Mail: selmadeniz@hakkari.edu.tr; 3 Department of Chemistry, Faculty of Science, İnönü University, Malatya 44280, Turkey; 4 Department of Elementary Education, Faculty of Education, Adıyaman University, Adıyaman 02040, Turkey; E-Mail: nsireci@adiyaman.edu.tr

**Keywords:** Suzuki-Miyaura reaction, homocoupling, *N*-heterocyclic carbene, microwave, pyridine derivatives

## Abstract

A number of novel benzimidazole derivatives **1**–**4** were synthesized and the catalytic activity of these compounds in a catalytic system consisting of a benzimidazolium salt/Pd(OAc)_2_/K_2_CO_3_ were investigated in the Suzuki-Miyaura and Ullmann type homocoupling reactions under microwave irradiation. We obtained both cross coupling and homocoupling products of pyridine and some side products such as dimethylaminopyridine and unsubstituted pyridine.

## 1. Introduction

Palladium-catalyzed cross-coupling reactions have emerged as a powerful method in organic synthesis for the formation of carbon-carbon bonds [1]. Among them, the Suzuki-Miyaura reaction plays an important role in modern synthetic chemistry [2]. Both symmetrical and unsymmetrical biaryls can be prepared easily using this type of reactions. There are many reports on the Suzuki-Miyaura cross-coupling reaction in the literature [3,4,5]. On the other hand, there are also noteworthy reports on the Ullmann type homocoupling reaction of aryl halides using different catalysts other than the traditional copper. Most of them involve stoichiometric amounts of transition metal salts such as TiCl_4_, TiCl, Vo(OEt)Cl_2_, CoCl_2_, FeCl_3_, and Pd(OAc)_2_. Some of them also need very reactive phenyl lithium or phenyl magnesium halide as a homocoupling partner [6,7,8,9,10,11,12]. In the coupling reactions, the efficiency of the Pd-based catalyst is strongly dependent on the nature of the coordinated ligands, solvent and reactivity of the aryl halides [13]. Bulky groups on the coupling partner also affect the catalytic yield in the Suzuki-Miyaura cross-coupling reactions [14]. The phosphine-based ligands are generally used as a ligand in this type of homocoupling reactions [15,16,17,18,19,20,21,22,23,24]. However tertiary phosphine ligands usually require air-free handling to prevent ligand oxidation and their P-C bond is unstable at elevated temperature, and as a consequence higher phosphine concentrations are required [25]. Since the isolation of the first stable free carbene by Arduengo *et al.* in 1991, the chemistry of carbenes and their transition metal-complexes have been very popular research subject in the organic and organometallic chemistry. Due to strong σ-donor but poor π-acceptor abilities, low toxicity and their stability to air and moisture, NHC ligands are considered alternatives to phosphine ligand in metal complexes [26,27,28,29,30,31,32].

On the other hand, microwave irradiation has gained importance in organic synthesis due to an increased life time of catalysts thorough the elimination of the wall effects, homogenous and short time heating and energy saving [33]. We have also observed significant contributions of microwave heating in Suzuki-Miyaura and Mizoroki-Heck cross coupling reactions using homoaromatic coupling partners [34,35,36,37,38,39]. After these observations, we also focused on halopyridines as the heteroaromatic coupling partner to obtain pyridine derivatives. As known, the pyridine motif is a ubiquitous building block found in many biologically active compounds. For example, methoxatin displays antioxidant properties, acodazole has antimicrobial and antineoplastic properties, whereas nifuroquine and telithromycin are antibacterial agents that all contain substituted pyridine moieties [40]. Due to limited information about cross coupling of arylboronic acid with aryl pyridines in the literature, we wish to report on the base-palladium-NHC-catalyzed direct arylation at the 2 or 3 position of pyridines and homocoupling of halopyridines and formation of some side products such as aminopyridine, biphenyl and pyridine.

## 2. Results and Discussion

The preparation of 2- or 3-arylsubstituted pyridine derivatives is of great importance in the synthesis of natural products, pharmaceutical and advanced functional compounds [41,42,43,44,45,46]. In recent years, there was an increasing interest in the synthesis of bipyridine derivatives because they are used in several areas such as an intermediate in chemical engineering and drug synthesis, inductor, photosensitive reagent and among them 2,2'-bipyridine is also important metal catalyst ligand [47,48,49,50,51]. But most of the reported synthesis require a long reaction time and provide only moderate yields. To our knowledge there is only one report of a catalytic synthesis of bipyridine under microwave irradiation using a phosphine based ligand and there is no example of palladium NHC catalyzed direct arylation using halopyridines and phenylboronic acid and homocoupling of the halopyridines under microwave heating conditions [52].

**Scheme 1:**
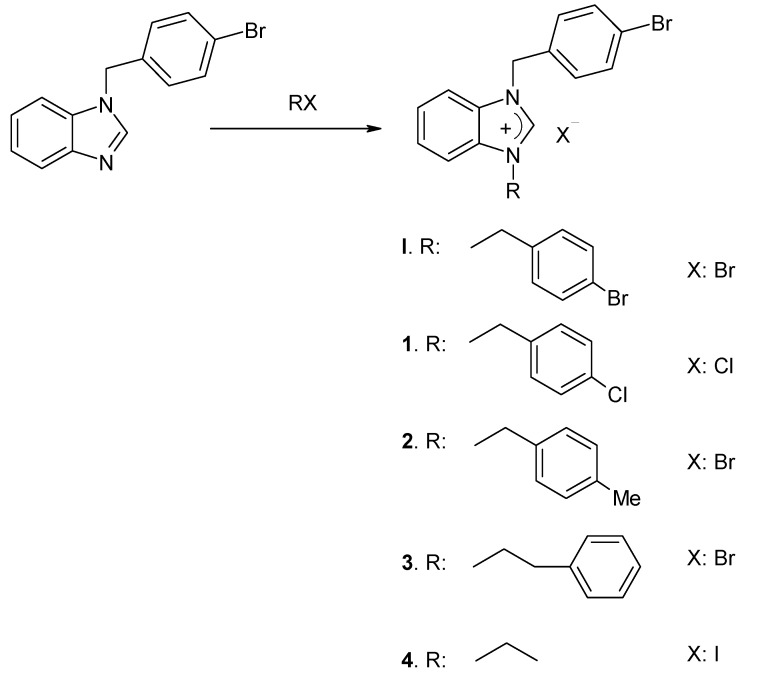
Synthesis of new benzimidazolium salts.

In this work the synthesis of the benzimidazole salts (**1**–**4**, and **I** [53]) from 1-(4-bromobenzyl)-benzimidazole [54] is shown in Scheme 1. Molecular structures of the new compounds **1**–**4** were identified by ^1^H, ^13^C-NMR, IR spectroscopic methods and elemental analysis. The NC*H*N proton signals for the benzimidazolium salts **1**–**4** were observed as singlets at δ = 10.23, 10.16, 9.77 and 9.88 ppm, respectively. The δ[^13^C{^1^H}], N*C*HN in benzimidazolium salts **1**–**4** were observed at δ = 143.5, 143.2, 142.9 and 142.7 ppm, respectively. These values are in good agreement with other recently reported results [34,35,36,37]. IR data for the C=N band frequencies, υ_(C=N)_, for benzimidazolium salts **1**–**4** were observed at 1,559, 1,559, 1,567 and 1,558 cm^−1^, respectively.

In the present work 2-bromopyridine was chosen as a model coupling partner for both the Suzuki-Miyaura type C-C cross-coupling and the Ullmann type homocoupling reaction to determine the best solvent for these reactions. The values of other parameters such as base, temperature and catalyst loading true catalyst amount were based on our recently reported optimal parameters [35]. DMF/H_2_O (1:1) mixture was chosen as a best solvent for the both the Suzuki-Miyaura and the Ullmann type coupling reactions.

### 2.1. The Suzuki-Miyaura Type Coupling Reaction

The Suzuki-Miyaura reaction is one of the most versatile and utilized reactions for the selective construction of carbon-carbon bonds, in particular for the formation of biaryl and heterobiaryl derivatives [2,55].

In this work, we aimed to determine efficiencies of benzimidazolium salts **I** and **1**–**4** in the coupling reactions between 2- or 3-halopyridines and phenylboronic acid under optimized conditions. The solvent effects were investigated using 2-bromopyridine and phenylboronic acid in terms of the main product yield (Suzuki-Miyaura product) in EtOH/H_2_O (1:1) 57.6% (Table 1, entry 3), DMA/H_2_O (1:1) 63.4% (Table 1, entry 4), DMF/H_2_O (1:1) with a 70.0% (Table 1, entry 5). After these results, DMF/H_2_O (1:1) mixture was chosen as a best solvent system for the coupling reaction. The catalytic reactivity was also investigated in pure water as a green solvent, but the coupling product yield decreased drastically to 2% (Table 1, entry 2). Furthermore, the yield of the Suzuki-Miyaura coupling product was significantly decreased absence of the ligand in the catalytic system (Table 1, entry 1). Finally, we found that the use of 1 mmol % Pd(OAc)_2_, 2 mmol % of **I**, **1**–**4** and 2 mmol of K_2_CO_3_ in DMF/H_2_O (1.1) at 120 °C/300 W microwave heating given rise to the best conversation within 10 min. Apart from the expected Suzuki-Miyaura cross coupling products, some side products such as biphenyl, pyridine, 2,2'-bipyridine or 3,3'-bipyridine, and 2-dimethylaminopyridine were also obtained varying amounts from the reaction mixtures. All of the results for the Suzuki-Miyaura type coupling reactions with side products were given in Table 1. As can be expected, heteroaryl chloride was less reactive than heteroaryl bromide, and the coupling yieldd of 2- or 3-chloropyridines with phenylboronic acid (Table 1, entries 10–14, 20–24) were found to be lower than those of the corresponding heteroaryl bromides (Table 1, entries 6–9, 15–19). It is noteworthy that the coupling product yields of 3-halopyridines with phenylboronic acid were higher than with 2-halopyridines. This is also expected result for the electrophilic reaction of the palladium catalyst with halopyridines due to the fact their more active site for this reaction is the β (3) position (Table 1, entries 6–9 and 15–19; 10–14 and 20–24). In comparing the side products, we obtained very low levels of amination products from 2-halopyridines (Table 1, entries 1, 4–6, 8, 10 and 12). Contrary to the literature information [56,57], we have not obtained any amination product for 3-halopyridines because the most active site for the nucleophilic attack on pyridine is the 2-position. Similar amination of aryl(heteroaryl) halides with amides using an expensive base such as KO^t^Bu and a long reaction time, except without phenylboronic acid, was reported in the literature recently [56,57].

We also obtained some bipyridine side product (Table 1, entries 1, 3–14, 18 and 22) almost with similar reactivity of 2- or 3-halopyridines, but the yields are more than those of the amination. We have also observed both biphenyl and bare pyridine as side products in reactions of both 2- and 3-halopyridines with phenylboronic acid (Table 1, last 2 columns). It is remarkable that the steric and electronic properties of the benzimidazole ligands have an influence on the reactivity of arylboronic acid with halopyridines for the Suzuki-Miyaura type cross-coupling reactions and also side product yields. Among the benzimidazole ligands, compound **4** having only an ethyl group was found to be the less reactive for the Suzuki-Miyaura type reactions (Table 1, entries 9, 14, 19 and 24). From the results obtained, it can be concluded that, the substituted phenyl rings on both the nitrogen atoms of the benzimidazole scaffold also play an important role for the catalytic conversion due to π-electron richness.

**Table 1:** The Suzuki-Miyaura reactions of halopyridines and the side products.

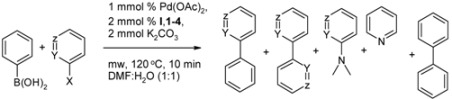									
Entry	X	Y	Z	Salt	% Yields				
					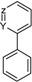	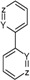	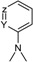	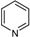	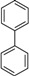
1 ^a^	Br	N	CH	**-**	40.3	13.8	1.1	0.6	17.2
2 ^b^	Br	N	CH	**2**	2.0	-	-	-	-
3 ^c^	Br	N	CH	**2**	57.6	5.3	-	-	15.6
4 ^d^	Br	N	CH	**2**	63.4	4.7	0.8	-	7.1
5 ^e^	Br	N	CH	**2**	70.0	22.0	0.3	0.7	7.0
6	Br	N	CH	**I**	65.0	23.7	0.7	0.7	9.9
7	Br	N	CH	**1**	67.0	27.2	-	1.6	4.2
8	Br	N	CH	**3**	72.5	17.8	0.6	1.3	7.8
9	Br	N	CH	**4**	66.8	18.8	-	-	4.4
10	Cl	N	CH	**I**	61.9	12.0	0.4	3.1	12.6
11	Cl	N	CH	**1**	64.1	14.5	-	9.1	6.1
12	Cl	N	CH	**2**	65.4	12.4	0.2	5.4	12.8
13	Cl	N	CH	**3**	66.1	12.5	-	4.4	9.0
14	Cl	N	CH	**4**	51.8	2.5	-	6.8	4.3
15	Br	CH	N	**I**	90.0	-	-	2.8	7.2
16	Br	CH	N	**1**	89.3	-	-	2.1	8.6
17	Br	CH	N	**2**	91.2	-	-	2.9	5.9
18	Br	CH	N	**3**	93.4	0.7	-	1.9	4.0
19	Br	CH	N	**4**	90.0	-	-	2.4	7.6
20	Cl	CH	N	**I**	66.6	-	-	2.5	23.8
21	Cl	CH	N	**1**	76.5	-	-	6.7	12.5
22	Cl	CH	N	**2**	70.1	0.7	-	2.0	16.4
23	Cl	CH	N	**3**	73.5	-	-	1.8	18.5
24	Cl	CH	N	**4**	58.0	-	-	4.8	9.2

*Reaction conditions*: 1.0 mmol halopyridine, 1.2 mmol phenylboronic acid, 2 mmol K_2_CO_3_, 1 mmol % Pd(OAc)_2_, 2 mmol % **I** or **1**–**4**, DMF (3 mL)-H_2_O (3 mL). Temperature ramped to 120 °C 3 min. Yields are based on aryl halide. Reactions were monitored by GC-MS. ^a^ No salt (ligand); ^b^ Solvent pure H_2_O; ^c^ EtOH/H_2_O (1:1); ^d^ DMA/H_2_O (1:1); ^e^ DMF/H_2_O (1:1).

### 2.2. The Ullmann Type Homocoupling Reaction

Efficiencies of benzimidazolium salts in homocoupling reactions were examined using 2- or 3-halopyridines. Similar to the Suzuki-Miyaura cross-coupling reaction, we first tried different solvent systems such as pure water (Table 2, entry 1), EtOH/H_2_O (1:1) (Table 2, entry 2) DMA/H_2_O (1:1) (Table 2, entry 3) and DMF/H_2_O (1:1) (Table 2, entry 4) due to find the best one. Among the solvent systems, DMF/H_2_O (1:1) was found the good solvent systems but the homocoupling yield was still low. Hence, the reaction time was increased to 30 min. Running the reaction for 30 min at DMF/H_2_O (1:1) mixture as a solvent we obtained reasonable homocoupling yield (Table 2, entry 5) and these parameters; 1 mmol % Pd(OAc)_2_, 2 mmol % of benzimidazolium salts and 2 mmol K_2_CO_3_ in DMF/H_2_O (1:1) at 120 °C/300 W microwave heating were chosen as an optimum reaction conditions. All of the results obtained using the optimum parameters for the Ullmann type coupling reactions, along with some side products, were given in Table 2. 

**Table 2:** The Ullmann type homocoupling reactions of halopyridines and side products.

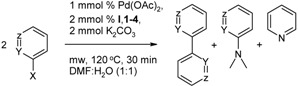							
Entry	X	Y	Z	Salt	% Yields		
					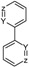	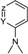	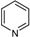
1 ^a^	Br	N	CH	**2**	1.3	-	-
2 ^b^	Br	N	CH	**2**	12.0	-	0.4
3 ^c^	Br	N	CH	**2**	43.5	9.9	-
4 ^d^	Br	N	CH	**2**	48.5	2.5	-
5	Br	N	CH	**I**	84.5	2.4	12.9
6	Br	N	CH	**1**	75.1	8.3	16.6
7	Br	N	CH	**2**	69.7	12.2	1.8
8	Br	N	CH	**3**	74.4	14.3	3.8
9	Br	N	CH	**4**	33.7	9.8	3.6
10	Cl	N	CH	**I**	64.3	0.6	34.4
11	Cl	N	CH	**1**	31.4	3.4	47.7
12	Cl	N	CH	**2**	46.5	5.1	23.2
13	Cl	N	CH	**3**	20.9	5.5	15.2
14	Cl	N	CH	**4**	8.0	5.5	3.7
15	Br	CH	N	**I**	15.0	-	85.0
16	Br	CH	N	**1**	5.2	-	94.8
17	Br	CH	N	**2**	6.6	-	93.4
18	Br	CH	N	**3**	2.3	-	97.7
19	Br	CH	N	**4**	-	-	62.2
20	Cl	CH	N	**I**	1.0	-	26.1
21	Cl	CH	N	**1**	-	-	40.6
22	Cl	CH	N	**2**	1.6	-	11.4
23	Cl	CH	N	**3**	1.0	-	10.6
24	Cl	CH	N	**4**	-	-	21.6

*Reaction conditions*: 2.0 mmol halopyridine, 2 mmol K_2_CO_3_, 1 mmol % Pd(OAc)_2_, 2 mmol % **I** or **1**–**4**, DMF (3 mL)- H_2_O (3 mL). Temperature ramped to 120 °C 3 min. Yields are based on aryl halide. Reactions were monitored by GC-MS. ^a^ Solvent pure H_2_O for 10 min; ^b^ Solvent EtOH/H_2_O (1:1) for 10 min; ^c^ DMA/H_2_O (1:1) for 10 min; ^d^ DMF/H_2_O (1:1) and for 10 min.

It is noteworthy that 2- or 3-chloropyridines (Table 2, entries 10–14, 20–24) are less reactive than the corresponding 2- or 3-bromopyridines (Table 2, entries 5–9, 15–19) in the Ullmann type homocoupling reactions, like the Suzuki-Miyaura coupling reactions. One of the side products, 3-dimethylaminopyridine was not observed in these reactions due to the less reactive site at the pyridine β-position. On the other hand, we obtained 2-dimethylaminopyridine as a side product in low yield (Table 2, entries 3–14). It is also noteworthy that we obtained bare pyridine from the homocoupling reactions with very high yield, particularly when using 3-bromopyridine as a homocoupling partner (Table 2, entries 15–19). Similar to the Suzuki-Miyaura cross coupling reactions, benzimidazole salt 4 with an ethyl substituent, showed the least activity in the homocoupling reactions, too (Table 2, entries 9, 14, 19 and 24). 

The coupling products of halopyridines are given in the literature as a Suzuki-Miyaura type or the Ullmann type coupling product only. In our work, we also determined some side products such as dimethylaminopyridine, and pyridine in addition of the main coupling products. To the best of knowledge, there is limited information about these types of products in the literature. Catalytic dimethylamino group transfer from some amides to aryl halides using Ni(phen)Cl_2_, and NHC-Pd(II)-Im complexes has been reported [56,57].

In conclusion, we prepared four new NHC precursor benzimidazolium salts containing substituted benzyl and phenethyl or ethyl groups **1**–**4**. The use of a palladium catalyst system including benzimidazolium salts/base and solvent in the Suzuki-Miyaura and the Ullmann type coupling reactions gives low to moderate yields under microwave-assisted conditions and relatively short reaction times compared with those reported in the literature [41,42,43,44,45,46,47,48,49,50,51].

## 3. Experimental

### 3.1. General Procedures

All of using starting chemicals were supplied commercially by Merck, Fluka or Aldrich Chemical Co. All catalytic activity experiments were carried out in a microwave oven system manufactured by Milestone (Milestone Start S Microwave Labstation for Synthesis) under aerobic conditions. Both ^1^H-NMR (300.13 MHz) and ^13^C-NMR (75.47 MHz) spectra were determined using a Bruker DPX-300 high-performance digital FT-NMR spectrometer. FT-IR spectra were recorded on a Perkin-Elmer spectrophotometer in the range 4,000–400 cm^−1^. Elemental analyses were performed by LECO CHNS-932 elemental analyzer. Melting points were recorded using an Electrothermal 9200 melting point apparatus, and are uncorrected.

GC-MS analysis were performed on an Agilient 6890 N GC and 5973 Mass Selective Detector system using by an HP-Innowax column of 60 m length, 0.25 mm diameter and 0.25 μm film thickness. GC-MS parameters for the S-M and homocoupling reactions were as follows: initial temperature 60 °C; initial time, 15 min; temperature ramp 1.30 °C/min; final temperature, 200 °C; ramp 2, 20 °C/min; final temperature 230 °C; total run time 72 min; injector port temperature 250 °C; detector temperature 250 °C; injection volume, 1.0 μL; carrier gas, helium; mass range between *m/z* 50–550.

All coupling product yields are based on aryl halides and determined as follows: the percent of the aryl halides were determined from the aryl halide chromatograms using the normalized peak areas method taken at the optimized chromatographic conditions. After completion of the reaction, the % coupling products were determined based on aryl halides. 1-(4-Bromobenzyl)benzimidazole [54] and its salt numbered **I** [53] were prepared according to literature methods. The syntheses procedures of new salts **1**–**4** are given below.

*Synthesis of 1-(4-bromobenzyl)-3-(4-chlorobenzyl)benzimidazolium chloride* (**1**). A mixture of 1-(4-bromobenzyl)benzimidazole (2.00 g, 6.96 mmol) and 4-chlorobenzyl chloride (1.14 g, 7.08 mmol) in DMF (5 mL) was refluxed for 4 h. After mixture was cooled solvent was removed under reduced pressure. The precipitate was crystallized from EtOH/Et_2_O (1:1) (30 mL). The product was obtained as white colored crystals. Yield: 2.28 g, 73%; mp 249–250 °C, IR, υ_(C=N)_ = 1559 cm^−1^. Anal. found: C, 55.87; H, 3.48; N, 5.91%. Calcd for C_21_H_17_N_2_BrCl_2_ (448.18): C, 56.28; H, 3.82; N, 6.25%. ^1^H-NMR (DMSO-d_6_) δ 5.83 (s, 2H, C*H_2_*C_6_H_4_Br), 5.84 (s, 2H, C*H_2_*C_6_H_4_Cl), 7.49-8.00 (m, 12H, Ar-*H*), 10.23 (s, 1H, NC*H*N). ^13^C-NMR (DMSO-d_6_) δ 49.8 (*C*H_2_C_6_H_4_Br and *C*H_2_C_6_H_4_Cl), 114.5, 122.6, 127.3, 129.5, 130.9, 131.2, 131.5, 132.4, 133.3, 133.8, 134.0 (Ar-*C*), 143.5 (N*C*HN). The compounds **2**, **3**, and **4** were similarly prepared from 1-(4-bromobenzyl)benzimidazole and the appropriate alkyl halides.

*1-(4-Bromobenzyl)-3-(4-methylbenzyl)benzimidazolium bromide* (**2**). Yield: 2.57 g, 78%; mp 255–256 °C, IR, υ_(C=N)_ = 1559 cm^−1^. Anal. found: C, 56.19; H, 4.34; N, 5.80%. Calcd for C_22_H_20_N_2_Br_2_ (472.21): C, 55.96; H, 4.27; N, 5.93%. ^1^H-NMR (DMSO-d_6_) δ 2.30 (s, 3H, CH_2_C_6_H_4_C*H_3_*); 5.77 (s, 2H, C*H_2_*C_6_H_4_CH_3_), 5.82 (s, 2H, C*H_2_*C_6_H_4_Br), 7.23–8.01 (m, 12H, Ar-*H*), 10.16 (s, 1H, NC*H*N). ^13^C-NMR (DMSO-d_6_) δ 21.2 (CH_2_C_6_H_4_*C*H_3_), 49.8 (*C*H_2_C_6_H_4_CH_3_), 50.4 (*C*H_2_C_6_H_4_Br), 114.5, 114.6, 122.6, 127.3, 128.8, 128.9, 130.0, 131.2, 131.3, 131.4, 131.5, 132.4, 133.8, 138.7 (Ar-*C*), 143.2 (N*C*HN). 

*1-(4-Bromobenzyl)-3-(2-phenylethyl)benzimidazolium bromide* (**3**). Yield: 2.83 g, 86%; mp 144–145 °C, IR, υ_(C=N)_ = 1567 cm^−1^. Anal. found: C, 55.39; H, 4.44; N, 5.74%. Calcd for C_22_H_20_N_2_Br_2_ (472.21): C, 55.96; H, 4.27; N, 5.93%. ^1^H-NMR (DMSO-d_6_) δ 3.27 (t, *J* = 7.2 Hz, 2H, CH_2_C*H_2_*C_6_H_5_), 4.82 (t, *J* = 7.2 Hz, 2H, C*H_2_*CH_2_C_6_H_5_), 5.72 (s, 2H, C*H_2_*C_6_H_4_Br), 7.17–8.13 (m, 13H, Ar-*H*), 9.77 (s, 1H, NC*H*N). ^13^C-NMR (DMSO-d_6_) δ 34.8 (CH_2_*C*H_2_C_6_H_5_), 48.4 (*C*H_2_CH_2_C_6_H_5_), 49.5 (*C*H_2_C_6_H_4_Br), 114.3, 114.5, 122.5, 127.2, 127.3, 127.4, 129.1, 129.3, 130.9, 131.1, 131.6, 132.3, 133.8, 137.3 (Ar-*C*), 142.9 (N*C*HN).

*1-(4-Bromobenzyl)-3-ethylbenzimidazolium iodide* (**4**). Yield: 2.07 g, 67%; mp 148–150 °C, IR, υ_(C=N)_ = 1558 cm^−1^. Anal. found: C, 44.03; H, 3.88; N, 6.04%. Calcd for C_16_H_16_N_2_BrI (443.12): C, 43.37; H, 3.64%; N, 6.32. ^1^H-NMR (DMSO-d_6_) δ 1.57 (t, *J* = 7.2 Hz, 3H, CH_2_C*H_3_*), 4.54 (q, *J* = 7.2 Hz, 2H, C*H_2_*CH_3_), 5.76 (s, 2H, C*H_2_*C_6_H_4_Br), 7.48-8.13 (m, 8H, Ar-*H*), 9.88 (s, 1H, NC*H*N). ^13^C-NMR (DMSO-d_6_) δ 14.5 (CH_2_*C*H_3_), 42.8 (*C*H_2_CH_3_), 49.7 (*C*H_2_C_6_H_4_Br), 114.3, 114.4, 122.5, 127.1, 127.2, 131.1, 131.3, 131.7, 132.3, 133.8 (Ar-*C*), 142.7 (N*C*HN).

### 3.2. General Procedure for the Suzuki-Miyaura and the Ullmann Type Homocoupling Reactions

#### 3.2.1. The Suzuki-Miyaura Reaction

Pd(OAc)_2_ (1 mmol %), benzimidazole salt (**I**, **1**–**4**) (2 mmol %), halopyridine (1 mmol), phenylboronic acid (1.2 mmol), K_2_CO_3_ (2 mmol) and mixture of solvent, water (3 mL)-DMF (3mL) were added in apparatus of microwave equipment in aerobic conditions. The mixture was heated at 120 °C, by microwave irradiation (300 Watt) for 10 min. At the end of reaction, the mixture extracted by ethyl acetate/*n*-hexane (1:5), filtered through 3 cm length column of silica gel. The solution was given to GC-MS equipment. The yields were determined by GC-MS based on halopyridine using the normalized peak areas method.

#### 3.2.2. Ullmann Type Homocoupling Reaction

Pd(OAc)_2_ (1 mmol %), benzimidazole salt **I**, **1**–**4** (2 mmol %), halopyridine (2 mmol), K_2_CO_3_ (2 mmol) and mixture of solvent, water (3 mL)-DMF (3 mL) were added in apparatus of microwave equipment in aerobic conditions. The mixture was heated at 120 °C, by microwave irradiation (300 Watt) for 30 min. After competition of the reaction, work-ups similar to those of the Suzuki-Miyaura reactions were followed to obtain the appropriate products.

## 4. Conclusions

In summary, we have synthesized several novel benzimidazolium salts through nucleophlic substitution reactions. Catalytic studies were done using catalytic systems consisting of Pd(OAc)_2_/benzimidazolium salt and K_2_CO_3_ for the Suzuki-Miyaura cross coupling and the Ullmann type homocoupling reactions of 2- or 3-halopyridines. We also obtained some side products such as dimethylaminopyridine dervivatives and pyridine with low yield in both the coupling reactions under microwave irradiation.

## Supplementary Materials

NMR spectra of the new compounds are available free of charge via the internet at http://www.mdpi.com/1420-3049/18/4/3712/s1.

## References

[1] Negishi E., de Meijere A.  (2002). Handbook of Organopalladium Chemistry for Organic Synthesis.

[2] Miyaura N., Suzuki A. (1995). Palladium-catalyzed cross-coupling reactions of organoboron compounds. Chem. Rev..

[3] Suzuki A. (1999). Recent advances in the cross-coupling reactions of organoboron derivatives with organic electrophiles, 1995–1998. J. Organomet. Chem..

[4] Fortman G.C., Nolan S.P. (2011). *N*-Heterocyclic carbene (NHC) ligands and palladium in homogeneous cross-coupling catalysis: a perfect union. Chem. Soc. Rev..

[5] Noël T., Buchwald S.L. (2011). Cross-coupling in flow. Chem. Soc. Rev..

[6] Inoue A., Kitagawa K., Shinokubo H., Oshima K. (2000). Simple and efficient TiCl_4_-mediated synthesis of biaryls via arylmagnesium compounds. Tetrahedron.

[7] McKillop A., Elsom L.F., Taylor E.C. (1968). Thallium in organic synthesis III. Coupling of aryl and alkyl Grignard reagents. J. Am. Chem. Soc..

[8] Ishikawa T., Ogawa A., Hirao T. (1998). Oxovanadium(V)-induced oxidative coupling of organolithium and -magnesium compounds. Organometallics.

[9] Kharasch M.S., Fields E.K. (1941). Factors determining the course and mechanism of Grignard reactions. IV. The effect of metallic halides on the reaction of aryl Grignard reagents and organic halides. J. Am. Chem. Soc..

[10] Nagaki A., Uesugi Y., Tomida Y., Yoshida J. (2011). Homocoupling of aryl halides in flow: Space integration of lithiation and FeCl_3_ promoted homocoupling. Beilstein J. Org. Chem..

[11] Bergeron-Brlek M., Giguère D., Shiao T.C., Saucier C., Roy R. (2012). Palladium-catalyzed Ullmann-type reductive homocoupling of iodoaryl glycosides. J. Org. Chem..

[12] Lei A.W., Srivastava M., Zhang X.M. (2002). Transmetalation of palladium enolate and its application in palladium-catalyzed homocoupling of alkynes: A room-temperature, highly efficient route to make diynes. J. Org. Chem..

[13] Del Zotto A., Amoroso F., Baratta W., Rigo P. (2009). Very fast Suzuki-Miyaura reaction catalyzed by Pd(OAc)_2_ under aerobic conditions at room temperature in EGME/H_2_O. Eur. J. Org. Chem..

[14] Zhang H., Kwong F.Y., Tian Y., Chan K.S. (1998). Base and cation effects on the Suzuki cross-coupling of bulky arylboronicacid with halopyridines: Synthesis of pyridylphenols. J. Org. Chem..

[15] Nadri S., Azadi E., Ataei A., Joshaghani M., Rafiee E. (2011). Investigation of the catalytic activity of a Pd/biphenyl-based phosphine system in the Ullmann homocoupling of aryl bromides. J. Organomet. Chem..

[16] Joshaghani M., Faramarzi E., Rafiee E., Daryanavard M., Xiao J., Baillie C. (2007). Highly efficient Suzuki coupling using moderately bulky tolylphosphine ligands. J. Mol. Catal. A Chem..

[17] Joshaghani M., Daryanavard M., Rafiee E., Xiao J., Baillie C. (2007). A highly efficient catalyst for Suzuki coupling of aryl halides and bromoarylphosphine oxides. Tetrahedron Lett..

[18] Adamo C., Amatore C., Ciofini I., Jutand A., Lakmini H. (2006). Mechanism of the palladium-catalyzed homocoupling of arylboronic acids: Key involvement of a palladium peroxo complex. J. Am. Chem. Soc..

[19] Billingsley K.L., Anderson K.W., Buchwald S.L. (2006). A highly active catalyst for Suzuki-Miyaura cross-coupling reactions of heteroaryl compounds. Angew. Chem. Ind. Ed. Engl..

[20] Seganish W.M., Mowery M.E., Riggleman S., DeShong P. (2005). Palladium-catalyzed homocoupling of aryl halides in the presence of floride. Tetrahedron.

[21] Wolfe J.P., Tomori H., Sodighi J.P., Yin J.J., Buchwald S.L. (2000). Simple, efficient catalyst system for the palladium-catalyzed amination of aryl chlorides, bromides, and triflats. J. Org. Chem..

[22] Iranpoor N., Firouzabadi H., Azadi R. (2008). Imidazolium-based phosphinite ionic liquid (IL-OPPh_2_) as Pd ligand and solvent for selective dehalogenation or homocoupling of aryl halides. J. Organomet. Chem..

[23] Zembayashi M., Tamao K., Yoshida J., Kumada M. (1977). Nickel-Phosphine complex-catalyzed homo coupling of aryl halides in the presence of zinc powder. Tetrahedron Lett..

[24] Iyoda M., Otsuka H., Sato K., Nisato N., Oda M. (1990). Homocoupling of aryl halides using nickel (II) complex and zinc in the presence of Et_4_NI. An efficient method for the synthesis of biaryls and bipyridines. Bull. Chem. Soc. Jpn..

[25] Peris E., Loch J.A., Mata J., Crabtre R.H. (2001). A Pd complex of a tridentate pincer CNC bis-carbene ligand as a robust homogenous Heck catalyst. Chem. Commun..

[26] Dawood K.M. (2007). Microwave-assisted Suzuki-Miyaura and Heck-Mizoroki cross-coupling reactions of aryl chlorides and bromides in water using stable benzothiazole-based palladium(II) precatalysts. Tetrahedron.

[27] Dallinger D., Kappe C.O. (2007). Microwave-assisted synthesis in water as solvent. Chem. Rev..

[28] Irfan M., Fuchs M., Glasnov T.N., Kappe C.O. (2009). Microwave-assisted Cross-Coupling and hydrogenation chemistry by using heterogeneous transition-metal catalysts: An evaluation of the role of selective catalyst heating. Chem. Eur. J..

[29] Brooker M.D., Cooper S.M., Hodges D.R., Carter R.R., Wyatt J.K. (2010). Studies of microwave-enhanced Suzuki-Miyaura vinylation of electron-rich sterically hindered substrates utilizing potassium vinyltrifluoroborate. Tetrahedron Lett..

[30] Martins D.L., Alvarez H.M., Aguiar L.C.S. (2010). Microwave-assisted Suzuki reaction catalyzed by Pd(0)-PVP nanoparticles. Tetrahedron Lett..

[31] Hajipour A.R., Karami K., Tavakoli G. (2011). A comparative homocoupling reaction of aryl halides using monomeric orthopalladated complex of 4-methoxybenzoylmethylenetriphenylphosphorane under conventional and microwave irradiation conditions. Appl. Organometal. Chem..

[32] Gädda T.M., Kawanishi Y., Miyazawa A. (2012). Microwave-assisted Ullmann-type coupling reactions in alkaline water. Synth. Commun..

[33] Liu L.-J., Wang F., Shi M. (2009). Elimination of an alkyl group from imidazolium salts: Imidazole-coordinated dinuclear monodentate NHC-palladium complexes driven by self-assembly and their application in the Heck reaction. Eur. J. Inorg. Chem..

[34] Yılmaz Ü., Şireci N., Deniz S., Küçükbay H. (2010). Synthesis and microwave-assisted catalytic activity of novel bis-benzimidazole salts bearing furfuryl and thenyl moieties in Heck and Suzuki cross-coupling reactions. Appl. Organometal. Chem..

[35] Yılmaz Ü., Küçükbay H., Şireci N., Akkurt M., Günal S., Durmaz R., Tahir M.N. (2011). Synthesis, microwave-promoted catalytic activity in Suzuki-Miyaura cross-coupling reactions and antimicrobial properties of novel benzimidazole salts bearing trimethylsilyl group. Appl. Organometal. Chem..

[36] Küçükbay H., Şireci N., Yılmaz Ü., Akkurt M., Yalçın Ş.P., Tahir M.N., Ott H. (2011). Synthesis, characterization and microwave-assisted catalytic activity of novel benzimidazole salts bearing piperidine and morpholine moieties in Heck cross-coupling reactions. Appl. Organometal. Chem..

[37] Küçükbay H., Şireci N., Yılmaz Ü., Deniz S., Akkurt M., Baktır Z., Büyükgüngör O. (2012). Synthesis, characterization, and microwave-promoted catalytic activity of novel benzimidazole reactions under aerobic conditions. Turk. J. Chem..

[38] Şireci N., Yılmaz Ü., Küçükbay H. (2010). Microwave assisted catalytic activity of some bis-5(6)-nitrobenzimidazole salts for Heck and Suzuki cross-coupling reactions. Asian J. Chem..

[39] Yılmaz Ü., Küçükbay H., Deniz S., Şireci N. (2013). Synthesis, characterization and microwave-promoted catalytic activity *of novel N-phe*n**ylbe**nz*im*idazolium salts in Heck-Mizoroki and Suzuki-Miyaura cross-coupling reactions under mild condtiyions. Molecules.

[40] Bensaid S., Doucet H. (2012). Palladium-catalysed direct arylation of heteroaromatics with functionalised bromopyridines. Tetrahedron.

[41] Liu C., Yang W. (2009). A fast and oxygen-promoted protocol for the ligand-free Suzuki reaction of 2-halogenated pyridines in aqueous media. Chem. Commun..

[42] Liu Y., Ye K., Fan Y., Song W., Wang Y., Hou Z. (2009). Amidinate-ligated iridium(III) bis(2-pyridyl)phenyl complex as an excellent phosphorescent material for electroluminescence devices. Chem. Commun..

[43] Mukhopadhyay S., Rothenberg G., Gitis D., Baidossi M., Ponde D.E., Sasson Y. (2000). Regiospecific cross-coupling of haloaryls and pyridine to 2-phenylpyridine using water, zinc, and catalytic palladium on carbon. J. Chem. Soc. Perkin Trans. 2.

[44] Tagata T., Nishida M. (2003). Palladium charcoal-catalyzed Suzuki-Miyaura coupling to obtain arylpyridines and arylquinolines. J. Org. Chem..

[45] Kudo N., Perseghini M., Fu G.C. (2006). A versatile method for Suzuki cross-coupling reactions of nitrogen heterocycles. Angew. Chem. Ind. Ed. Engl..

[46] Wen J., Qin S., Ma L.-F., Dong L., Zhang J., Liu S.-S., Duan Y.-S., Chen S.-Y., Hu C.-W., Yu X.-Q. (2010). Iron-Mediated Direct Suzuki-Miyaura Reaction: A New Method for the ortho-Arylation of Pyrrole and Pyridine. Org. Lett..

[47] Hennings D.D., Iwama T., Rawal V.H. (1999). Palladium-catalyzed (Ullmann-Type) homocoupling of aryl halides: A convenient and general synthesis of symmetrical biaryls via inter- and intramolecular coupling reactions. Org. Lett..

[48] França K.W.R., Navarro M., Leonel E., Durandetti M., Nedelec J.-Y. (2002). Electrochemical homocoupling of 2-bromomethylpyridines catalyzed by nickel complexes. J. Org. Chem..

[49] Cravotta G., Beggiato M., Penoni A., Palmisano G., Tollari S., Lévêque J.-M., Bonrath W. (2005). High-intensity ultrasound and microwave, alone or combined, promote Pd/C-catalyzed aryl-aryl couplings. Tetrahedron Lett..

[50] Tao X., Zhou W., Zhang Y., Dai C., Shen D., Huang M. (2006). Homocoupling of aryl bromides catalyzed by nickel chloride in pyridine. Chin. J. Chem..

[51] Park B.R., Kim K.H., Kim T.H., Kim J.N. (2011). Palladium-catalyzed benzoin-mediated redox process leading to biaryls from aryl halides. Tetrahedron Lett..

[52] Moore L.R., Vicic D.A. (2008). A heterogeneous-catalyst-based, microwave-assisted protocol for the synthesis of 2,2'-bipyridines. Chem. Asian. J..

[53] Mo J.-M., Ma Y.-G., Cheng Y. (2009). Synthesis of novel synthetic intermediates from the reaction of benzimidazole and triazole carbenes with ketenimines and their application in the construction of spiro-pyrroles. Org. Biomol. Chem..

[54] Wan Y., Wallinder C., Plouffe B., Beaudry H., Mahalingam A.K., Wu X., Johansson B., Holm M., Botoros M., Karlén A. (2004). Design, synthesis, and biological evaluation of the first selective nonpeptide AT2 receptor agonist. J. Med. Chem..

[55] Chanthavong F., Leadbeater N.E. (2006). The application of organic bases in microwave-promoted Suzuki coupling reactions in water. Tetrahedron Lett..

[56] Zhao J.K., Wang Y.G. (2002). A facile and efficient synthesis of *N,N*-dimethylarylamines from aryl bromides. Chin. Chem. Lett..

[57] Chen W.-X., Shao L.-X. (2012). N-Heterocyclic Carbene-Palladium(II)-1-Methylimidazole Complex Catalyzed Amination between Aryl Chlorides and Amides. J. Org. Chem..

