# Supplementary material for: Microwave Assisted Suzuki-Miyaura and Ullmann Type Homocoupling Reactions of 2- and 3-Halopyridines Using a Pd(OAc)2/Benzimidazolium Salt and Base Catalyst System
Source: Molecules. 2013 Mar 25;18(4):3712–24. doi: 10.3390/molecules18043712 (PMC6269664; doi:10.3390/molecules18043712)

Supportıng Materials

Figure S1. ^{1}H-NMR spectrum of compound **1**.

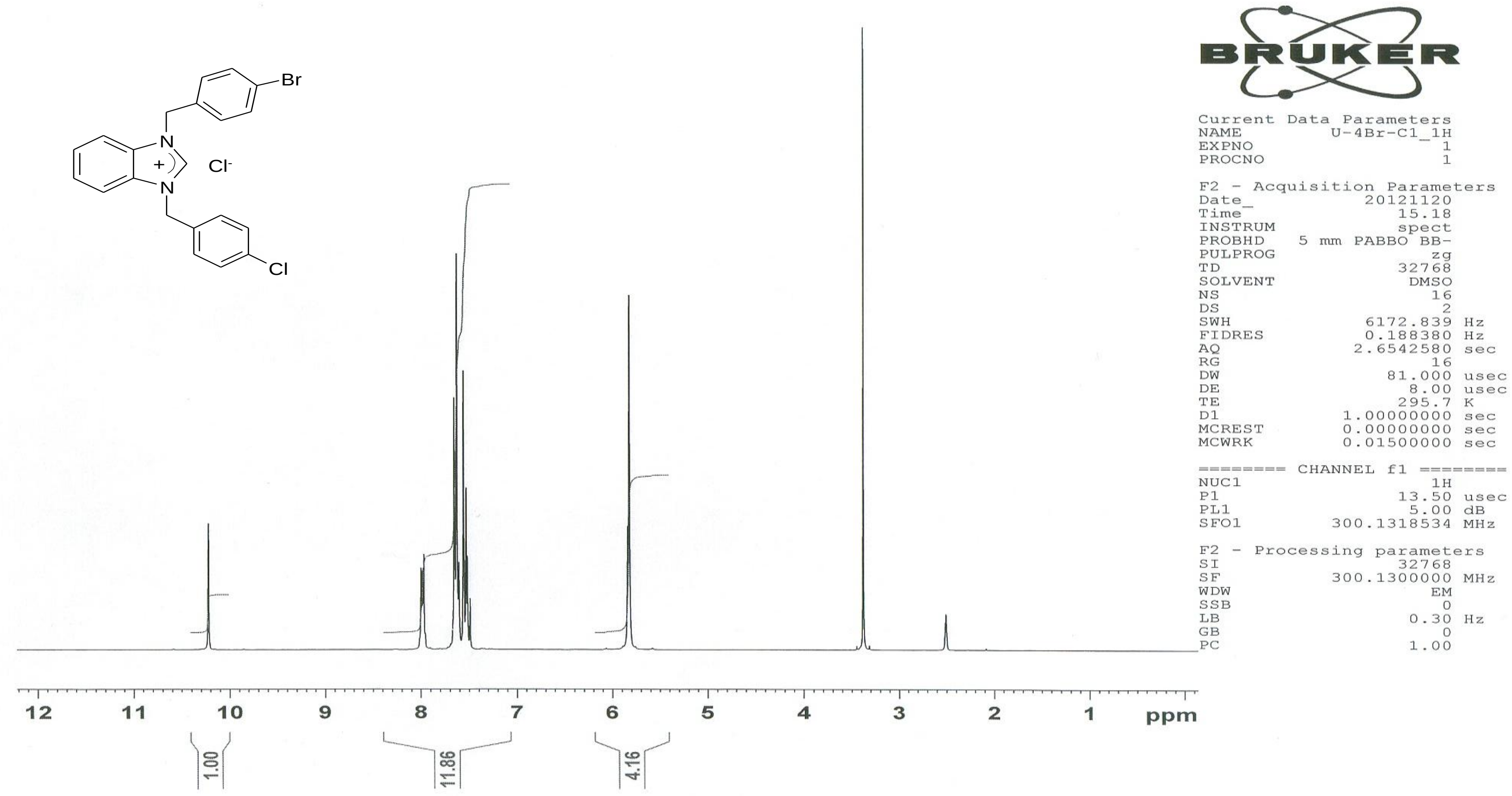

Figure S2. ^{13}C-NMR spectrum of compound **1**.

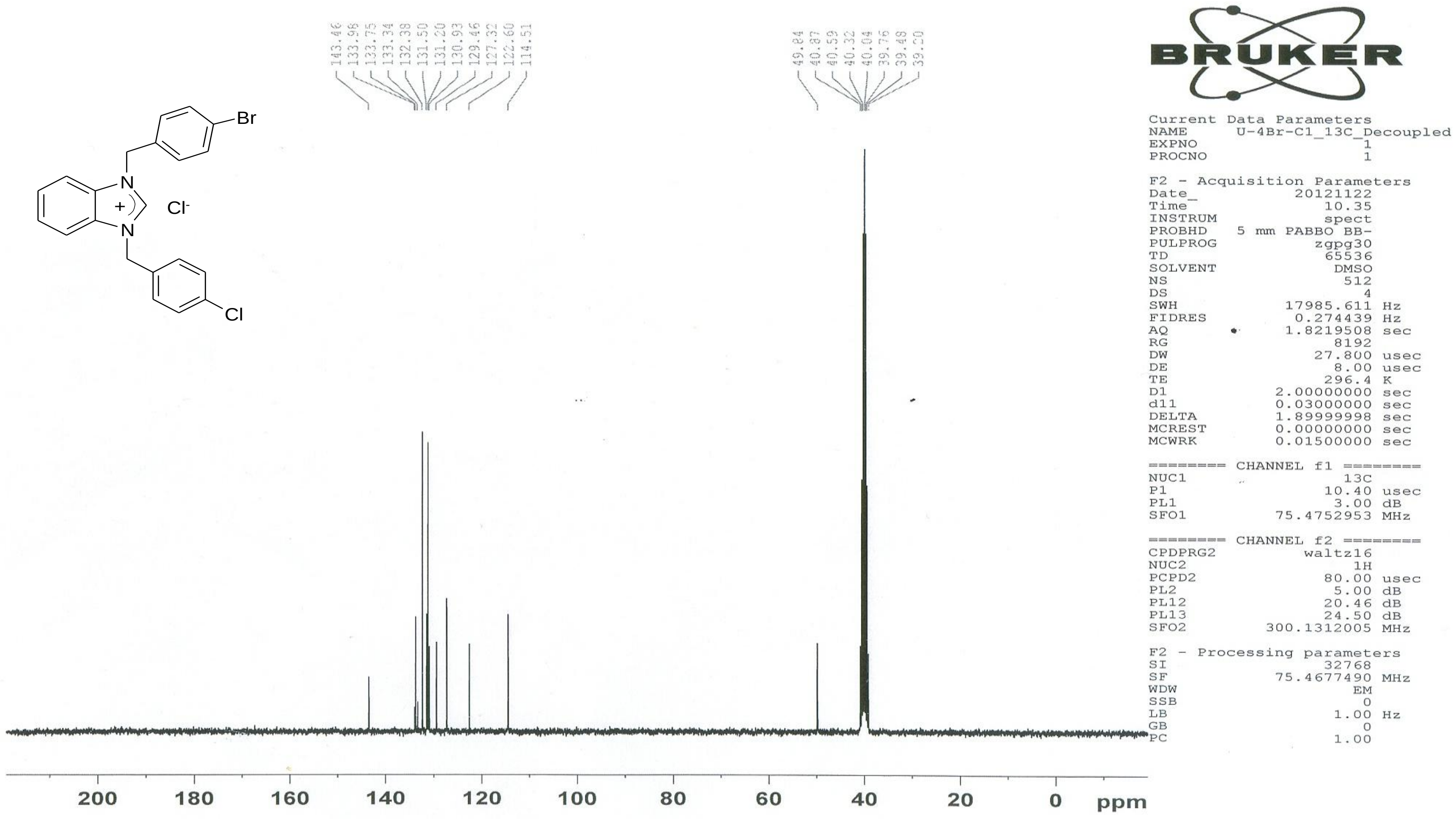

Figure S3. ^{1}H-NMR spectrum of compound **2**.

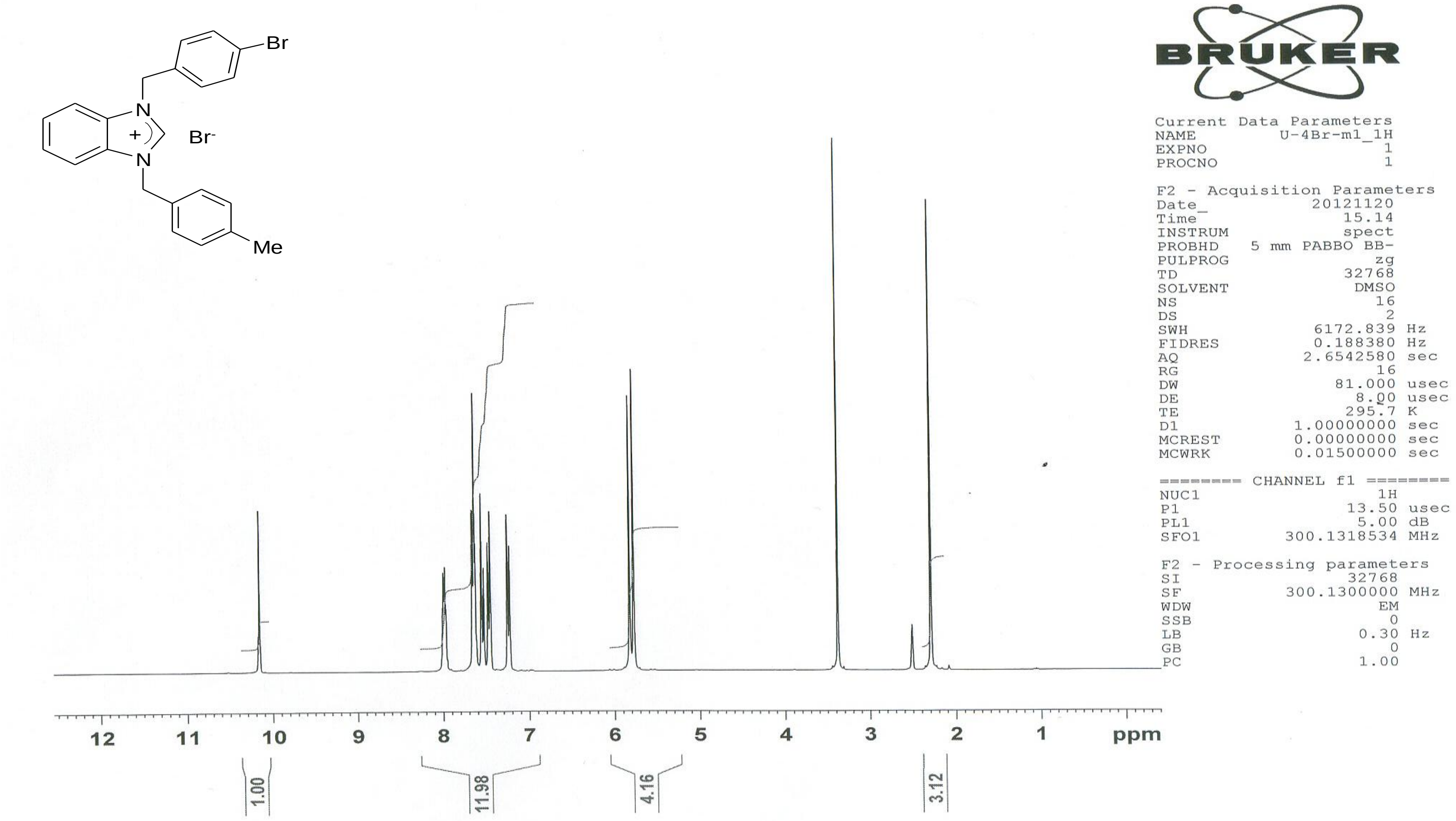

Figure S4. ^{13}C-NMR spectrum of compound **2**.

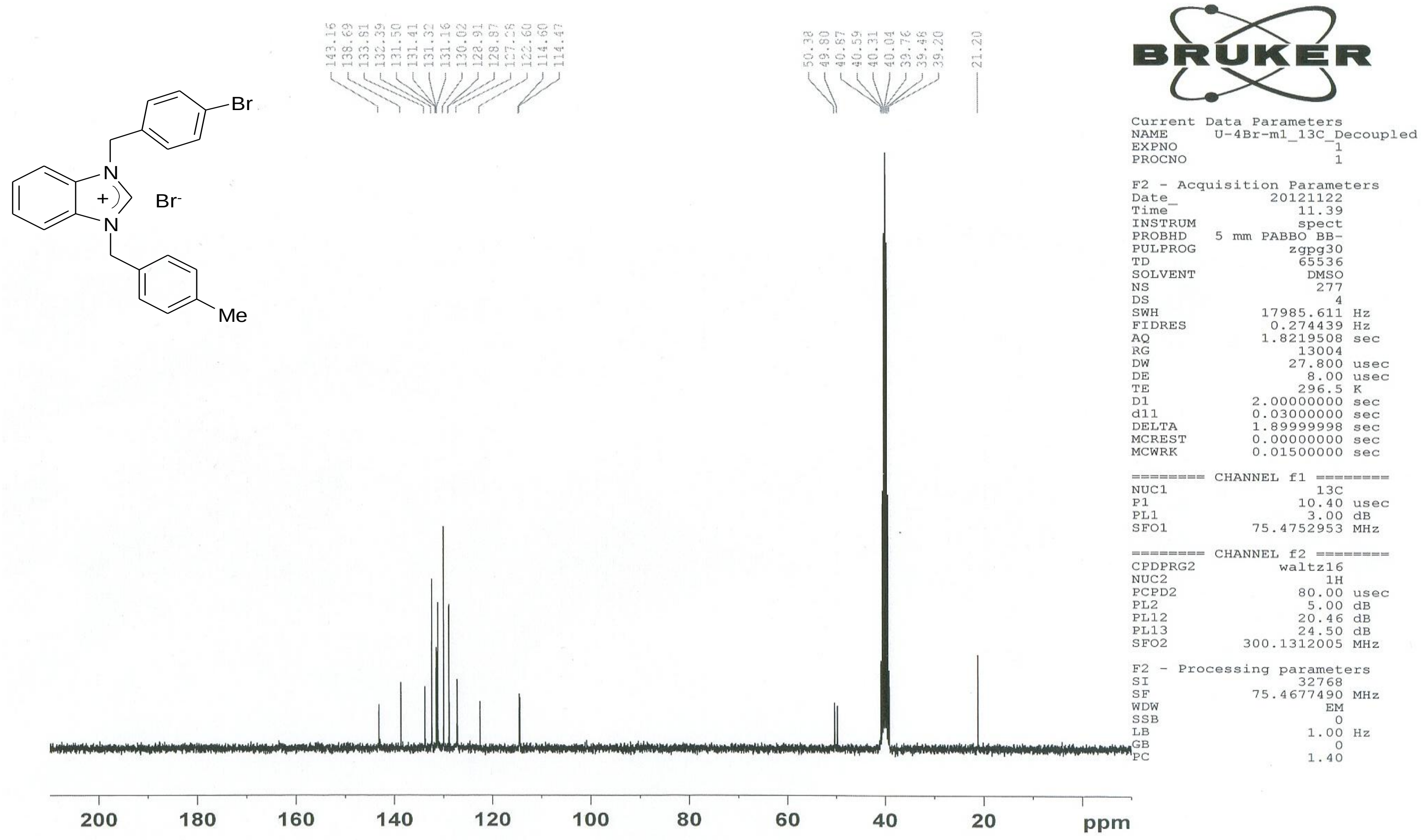

Figure S5. ^{1}H-NMR spectrum of compound **3**.

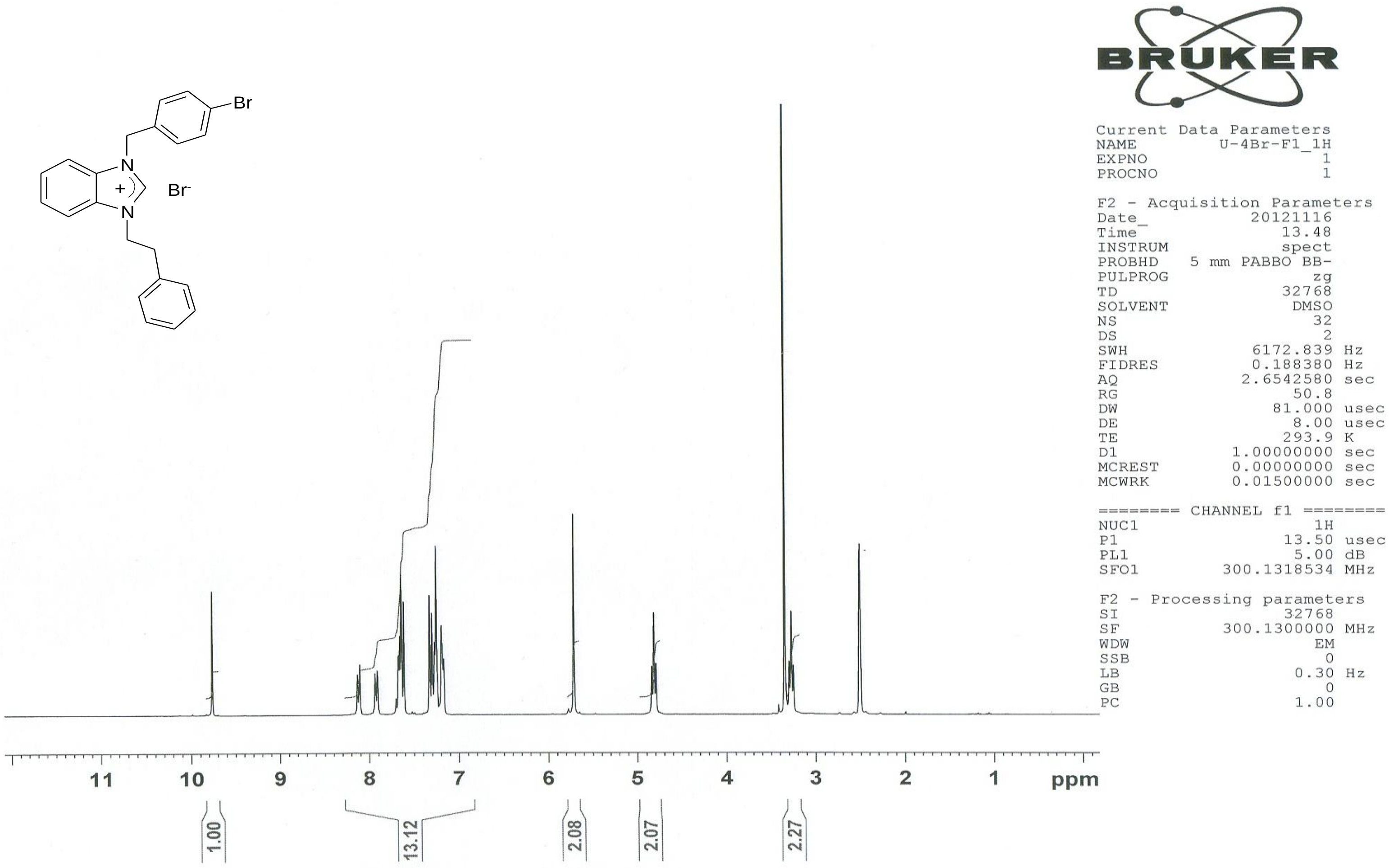

Figure S6. ^{13}C-NMR spectrum of compound **3**.

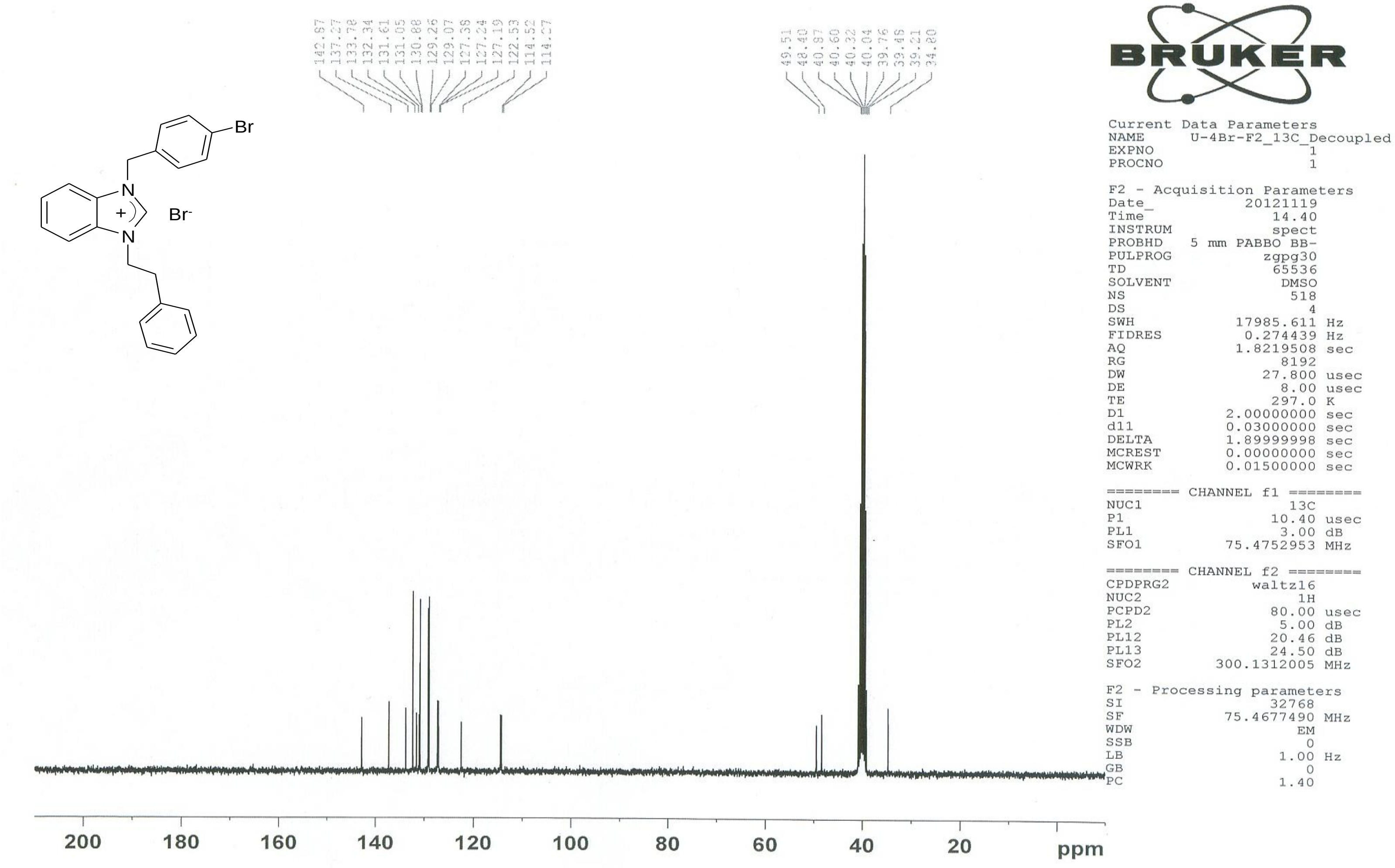

Figure S7. ^{1}H-NMR spectrum of compound **4**.

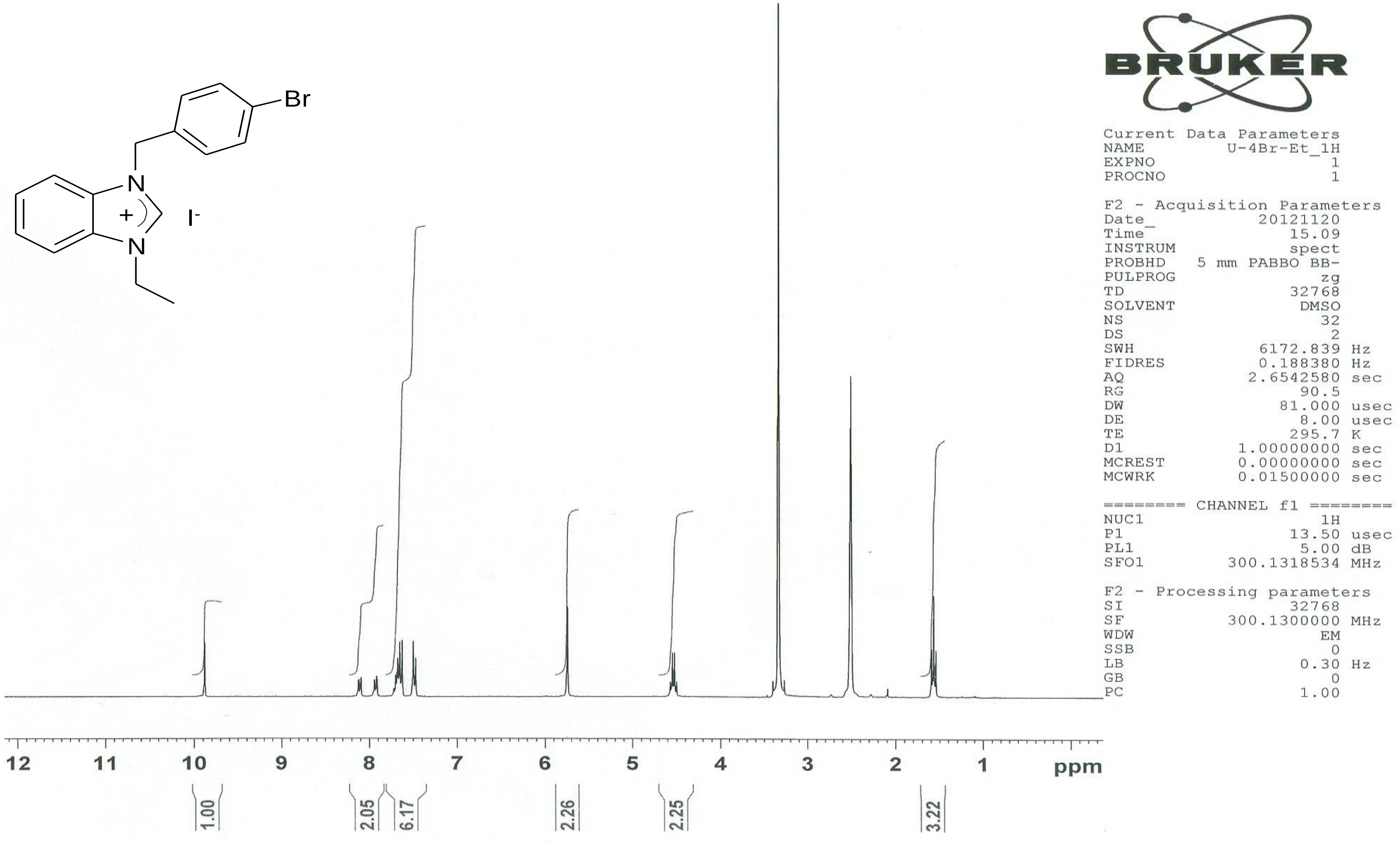

Figure S8. ^{13}C-NMR spectrum of compound **4**.

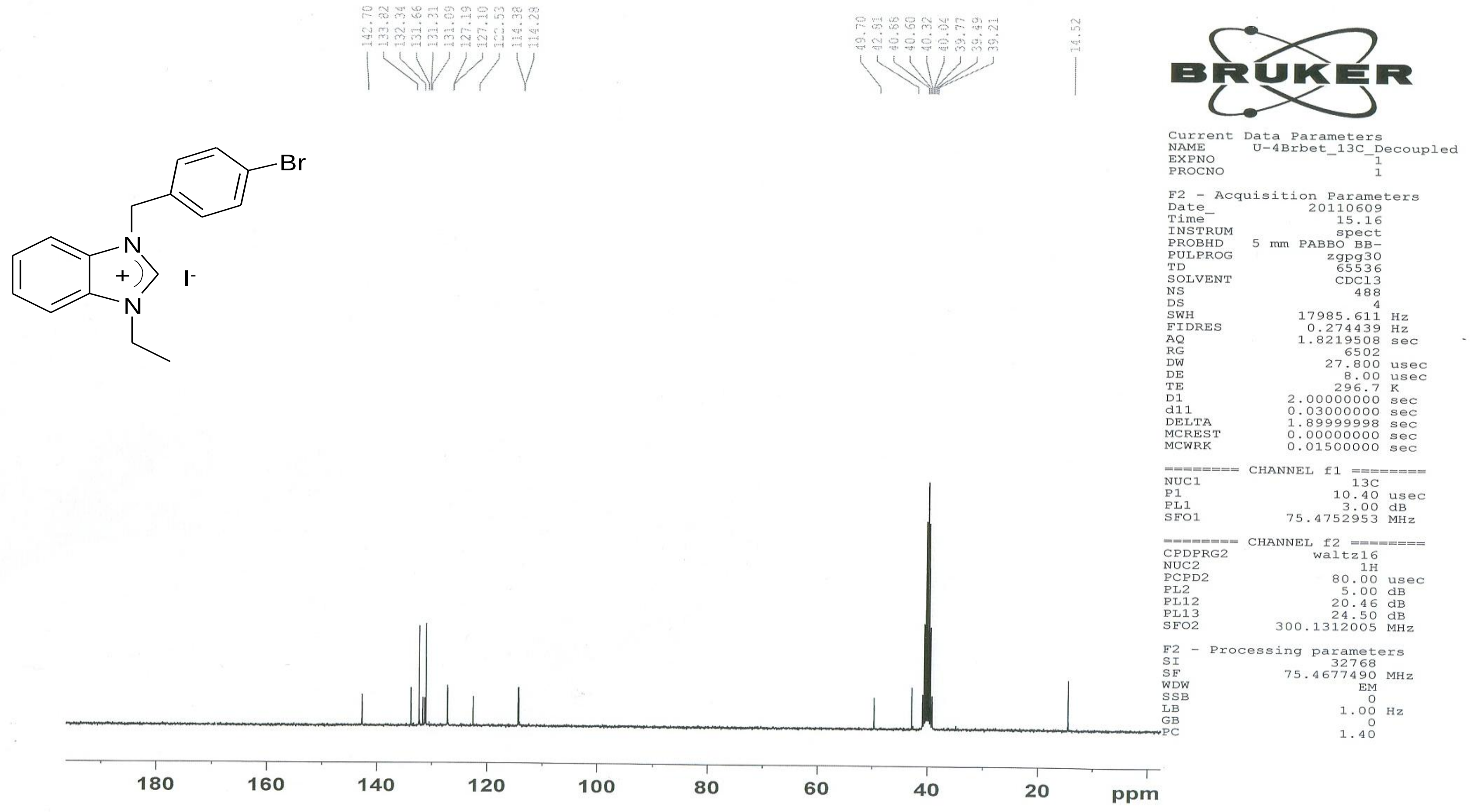

Supplement: Supplementary file 1 [file molecules-18-03712-s001.pdf]
